# Case Report: The value of contrast-enhanced ultrasound and contrast-enhanced computed tomography in the diagnosis of hepatic angiosarcoma

**DOI:** 10.3389/fonc.2023.1283544

**Published:** 2023-12-01

**Authors:** Feiqian Wang, Kazushi Numata, Hua Liang, Hiromi Tsuchiya, Litao Ruan, Mikiko Tanabe, Xiaofang Bai

**Affiliations:** ^1^ Ultrasound Department, The First Affiliated Hospital of Xi’an Jiaotong University, Xi’an, Shaanxi, China; ^2^ Gastroenterological Center, Yokohama City University Medical Center, Yokohama, Kanagawa, Japan; ^3^ Department of Pathology, The First Affiliated Hospital of Xi’an Jiaotong University, Xi’an, Shaanxi, China; ^4^ Division of Diagnostic Pathology, Yokohama City University Medical Center, Yokohama, Kanagawa, Japan

**Keywords:** hepatic angiosarcoma, contrast-enhanced ultrasound, contrast-enhanced computed tomography, diagnosis, image

## Abstract

**Background:**

Enhanced imaging techniques have the overwhelming advantages of being noninvasive and sensitive enough to evaluate the microcirculation of lesions, thus making them accurate in the diagnosis of hepatic lesions. Unfortunately, there is very little research on and knowledge of the imaging features of a rare cancerous condition: hepatic angiosarcoma (HA).

**Case summary:**

In this study, we retrospectively collected the data of six patients who underwent both contrast-enhanced ultrasound (CEUS) and contrast-enhanced computed tomography (CECT), and subsequently obtained a definitive histopathologic diagnosis of HA. We described the imaging appearances of HA by comparing CEUS and CECT images. Furthermore, we analyzed these imaging characteristics from the perspective of histopathology and tumorigenesis. The study included the largest number (six) of histopathologically confirmed HA patients who had received CEUS examinations to date.

**Conclusion:**

By offering readers comprehensive knowledge of contrast imaging, especially CEUS, in the diagnosis of HA, our study may reduce misdiagnosis and further improve treatment options.

## 1 Introduction

Hepatic angiosarcoma (HA) is a rare interstitial malignancy caused by endothelial dysplasia of the hepatic sinusoids. Because of HA’s low incidence [accounts for 2% of primary hepatic tumors (1)] and doctors’ limited experience in diagnosing it, it is easily misdiagnosed as hemangioma or liver cancer (1, 2) before a histopathological diagnosis is obtained. Among all imaging modalities, contrast-enhanced computed tomography (CECT) is very commonly used in the liver. CECT was considered a first-line imaging modality for the characterization of liver lesions (3) and for diagnosing HCC by all major clinical practice guidelines (4). In recent years, contrast-enhanced ultrasound (CEUS) has been increasingly used because of its well-known advantages, such as being cost-effective, easy to perform, immediately available, reproducible in real-time, and radiation-free (5). CEUS can be used in patients with claustrophobia or who have cardiac pacemakers. CEUS equipment, as opposed to the CT unit, can shift to the bedside in the intensive care unit (6). Nevertheless, little is known about the usefulness of CECT and especially CEUS in diagnosing HA because there are very few relevant published studies. To complicate matters, only one to three patients have previously been included in published CEUS studies (2, 7–10).

We considered noninvasive preoperative methods important to guide clinical decision-making concerning HA. Regarding diagnosis of HA, biopsy performance is not recommended as it carries a significant risk of inducing hemorrhage (11, 12). Herein, using detailed depiction and analysis of two popular contrast-specific imaging methods (CEUS and CECT) from six patients who have been definitively diagnosed with HA, we evaluated the diagnostic value of CEUS and CECT in the hope of reducing biopsy performance in the future. Regarding treatment, HA is an absolute contraindication for transplantation (13, 14). Therefore, we further hope to provide an imaging strategy to assist in the proper therapeutic management of HA.

## 2 Materials and methods

### 2.1 Data collection

The data of the six patients included in this study were retrospectively collected from the pathological diagnosis system of two institutions: the First Affiliated Hospital of Xi’an Jiaotong University in China, and the Yokohama City University Medical Center in Japan. The time period for the search was from January 2015 to June 2023, based on discharge diagnoses of “hepatic angiosarcoma” by searching the patients’ electronic medical records. We retrospectively collected general clinical data (patients’ gender, age, etiology, medical history, and the process of treatment), as well as data from laboratory examinations (tumor markers, liver function, etc.). Written consent was obtained from the eventually selected patients or their immediate families to publish the patients’ information. All data collection and diagnostic and therapeutic procedures performed with these patients were in accordance with the principles of the Declaration of Helsinki.

### 2.2 Radiological examination

CEUS and CECT data were collected from the Picture Archiving and Communication System of our two institutions. CEUS and CECT were carefully checked to have been performed within 1 month before biopsy.

A LOGIQ E9 US system (GE Healthcare, Milwaukee, WI, USA) or Resona 7 US System (Mindray, Shenzhen, China) equipped with native tissue harmonic grayscale imaging and CEUS function was used. Convex and microconvex transducers with frequencies of 1–6 and 2–5 MHz were used. The contrast agents, operating methods, and setting conditions of CEUS in the Chinese hospital and the Japanese hospital are different. Please see **Supplementary Table 1**. CEUS images were acquired during three contrast phases, consisting of an arterial phase (AP) (10–20 s to 30–50 s after contrast injection), portal venous phase (PP) (30–50 s to 120 s), and the last phase. The last phase is different between the Chinese hospital, which uses SonoVue (delayed phase, >120 s, until bubble disappearance around 4–6 min)s and the Japanese hospital, which uses Sonazoid (postvascular phase,>10 min, until approximately 30 min).

Two 256-slice CT scanners, namely the Philips Brilliance iCT from Medical Systems in Best, the Netherlands, and the Revolution CT from GE Healthcare in Milwaukee, WI, were utilized to conduct an enhanced abdominal CT scan. For all the patients, AP of CT scans was obtained at 30 s, while PP of CT scans was obtained at 60–70 s after the injection of contrast agent. For patients in the Japanese hospital, more detailed scans of the equilibrium phase (180 s after the injection) were acquired.

### 2.3 Histological diagnosis

All six patients underwent ultrasound (US)-guided percutaneous transhepatic biopsy. For multiple lesions, biopsies were performed on the largest lesion. Other than hematoxylin–eosin (HE) staining, immunohistochemical staining of the cluster of differentiation (CD) antigens CD34 and CD31 (endothelial cell markers), as well as Ki-67 (proliferation marker) and vimentin (the relative specific markers of mesenchymal cells and mesenchymal-derived tumors), were performed. Pathologists with more than 10 years of experience in liver pathology reviewed all the specimen slices. Because of the retrospective nature of this study, the type of immunohistochemistry was not specified by the authors of this study. It depended on the pathologists’ experience and preferences, as well as the requirements of the hepatologists who were in charge of the treatment of the patients. Therefore, the staining types for each specimen were slightly different.

## 3 Results

### 3.1 Clinical data

The general information of all six patients is displayed in **Supplementary Table 2**. All the patients were aged, with an average age of 65.3 years old (ranging from 58 to 74 years old). The chief complaints were mostly of epigastric pain which was, nonetheless, nonspecific. Personal medical history and family history were unremarkable, especially the history of exposure to chemical substances. The tumor marker alpha-fetoprotein (AFP) was within normal ranges. Other tumor markers such as carbohydrate antigen 125 (CA125) (1 out of 6, 16.7%), carbohydrate antigen 19-9 (CA19-9) (3 out of 6, 50%), and carcinoembryonic antigen (CEA) (3 out of 6, 50%) were only slightly elevated in a few patients. Hepatitis B and C serology were all negative. Five in six patients manifested abnormal hematological examination indices that indicated the likelihood of bleeding, abnormal coagulation, or anemia. All three patients from China received active treatment (patients No.1 and No.2 received chemotherapy while patient No.3 undertook transcatheter arterial chemoembolization). All three patients from Japan (patients No.4, No.5, and No.6) were treated by “best supportive care” only. They all died, with an average survival of 9.3 months, ranging from 2 to 20 months (**Supplementary Figure 1**).

### 3.2 Grayscale US and CEUS examination

**Figure 1:**
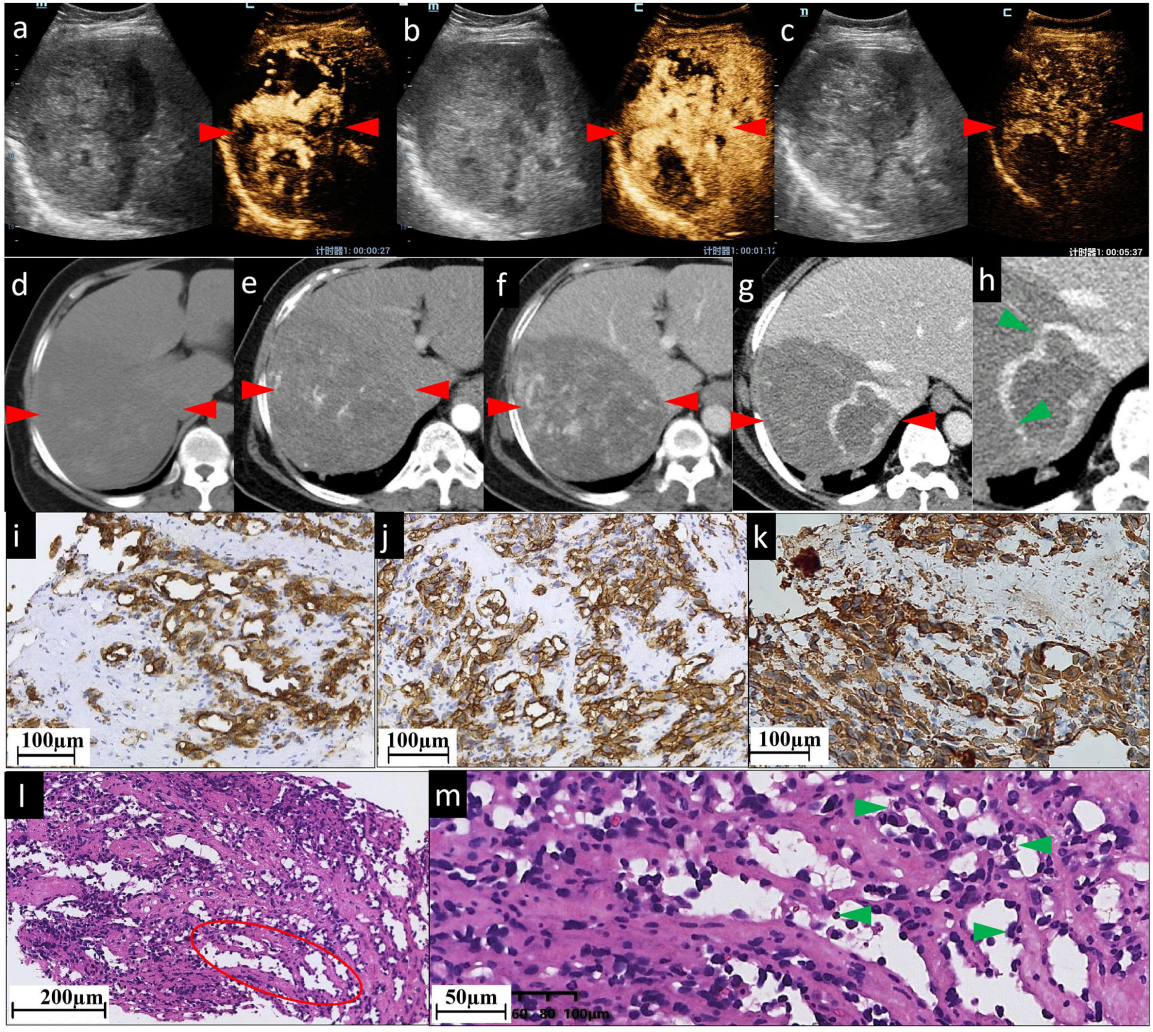
The CEUS and CECT images and histopathological picture of patient No. 1. **(A–C)** CEUS images show inhomogeneous patchy hyperenhancement in AP **(A)**, continuous hyperenhancement in PP **(B)** and hypoenhancement in delayed phase **(C)**. **(D)** In the right lobe of the liver, there is an approximately 103×133 mm slightly hypodense shadow. It has ill-defined borders, irregular shape, and inhomogeneous inner density. After being contrast-enhanced, the lesion shows moderate **(E)** and persistent **(F)** enhancement, with patchy marked enhancement in AP and progressive enhancement in PP. It is clearly visualized on a 1 mm thin section scanned PP image **(G)** and the local magnification image (**H**, green arrowheads); the right branch of the hepatic vein enters the lesion with a few filling defects (indicating the tumor has already invaded the right hepatic vein), while the right branch of portal vein enters the lesion with a natural course and a slightly brown border (not shown). In pictures of immunohistochemical staining, diffuse CD31 **(I)** and CD34 **(J)** expression suggest positive immunoreactivity in sinusoidal capillarization. **(K)** Diffuse vimentin expression shows a large number of mesenchymal cells. **(L, M)** are histopathological examinations with HE staining. The red ovoid area shows dilated vascular lumina anastomosed to each other. **(L)** shows spindled-shaped cells and epithelioid cells arranged in sheets with lacunae scattered between them. **(M)** shows that the dilated sinusoid-like lumina are lined with single- or multilayered atypical epithelial cells and spindle cells. Their nuclei are enlarged and hyperchromatic, displaying an abnormal shape (round or oval, with obvious nucleoli). Green arrowheads in **(M)** indicate atypical epithelial cells projecting into the lumen to form papillary structures. No obvious thrombosis is found in the sinuses. The red arrowheads seen in **(A–F)** indicate the border of the lesion.

**Figure 2:**
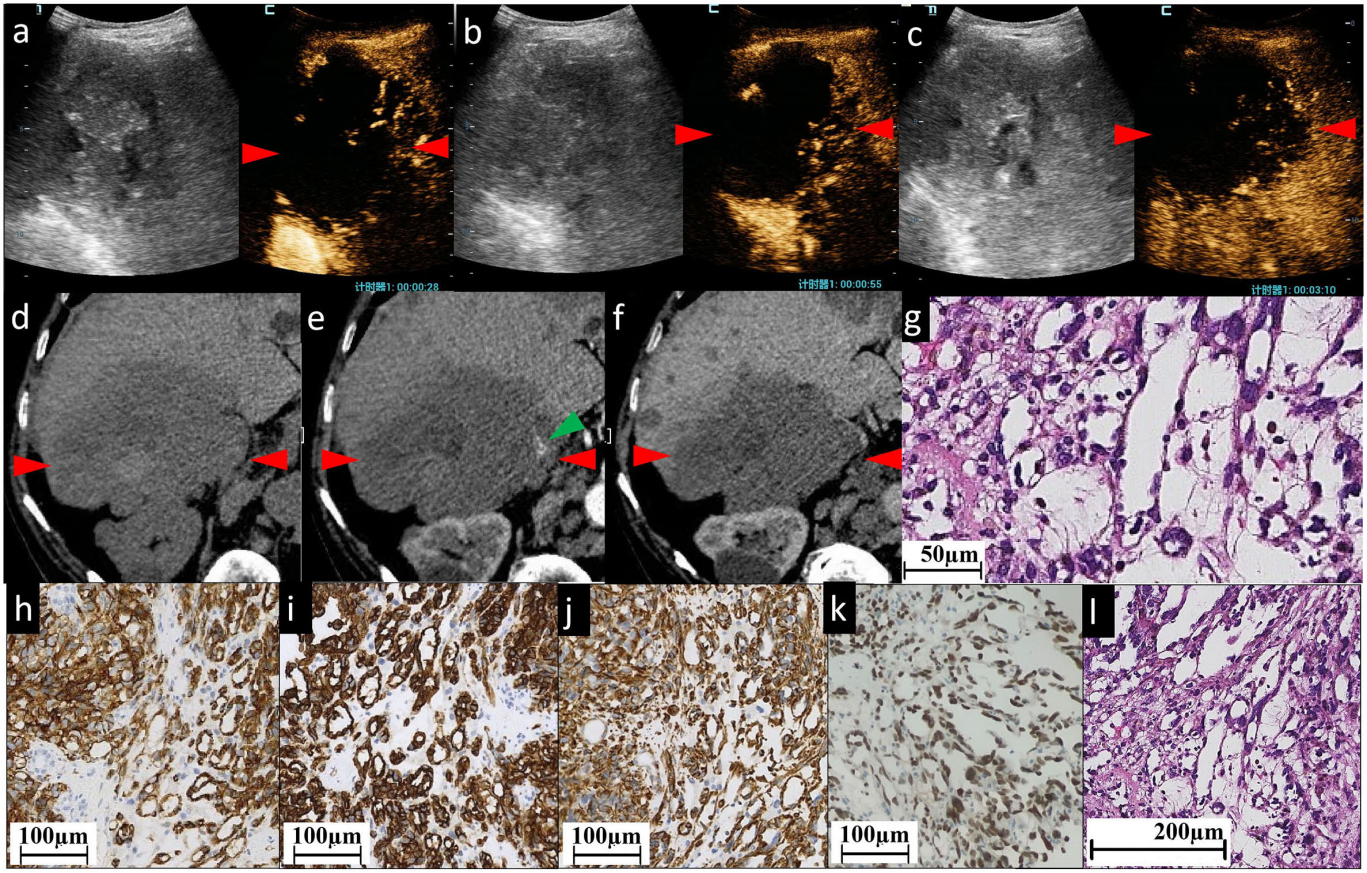
The CEUS and CECT images and histopathological picture of patient No. 2. **(A–C)** CEUS images show most areas of the lesion have perfusion defects of contrast agent, except some sparse, thin, linear hyperenhancement in AP **(A)**, continuous hypervascularity of these line-like structures in PP **(B)** and hypoenhancement in delayed phase **(C)**. Unenhanced CT image **(D)** reveals a heterogeneous low-density mass of approximately 102×90 mm in the right lobe of the liver, with an irregular shape and ill-defined boundary. **(E, F)** indicate CECT. The mass is characterized by slightly linear peripheral enhancement (green arrowhead) in AP **(E)** and the enhancement is persistent in PP **(F)**. Almost all areas of the lesion have no enhancement. **(G, L)** Histopathological examination with HE staining showing the structure of sinusoid-like lumina which are severely dilated, have complex constructions, and coincide with each other. Infiltrating spindle cell malignant tumors are surrounded by hepatocytes. Immunohistochemical staining of cytoplasm for vascular antigen CD31 **(H)** and CD34 **(I)** shows endothelial tumor cells differentiated in clusters and bundles. **(J)** Diffuse vimentin expression shows a large number of mesenchymal cells. Immunohistochemical staining for ERG (erythroblast transformation-specific (ETS)-related gene) **(K)** shows positive staining of the nucleus showing diffuse vascular endothelial differentiated tumor cells. The red arrowheads seen in **(A–F)** indicate the border of the lesion.

**Figure 3:**
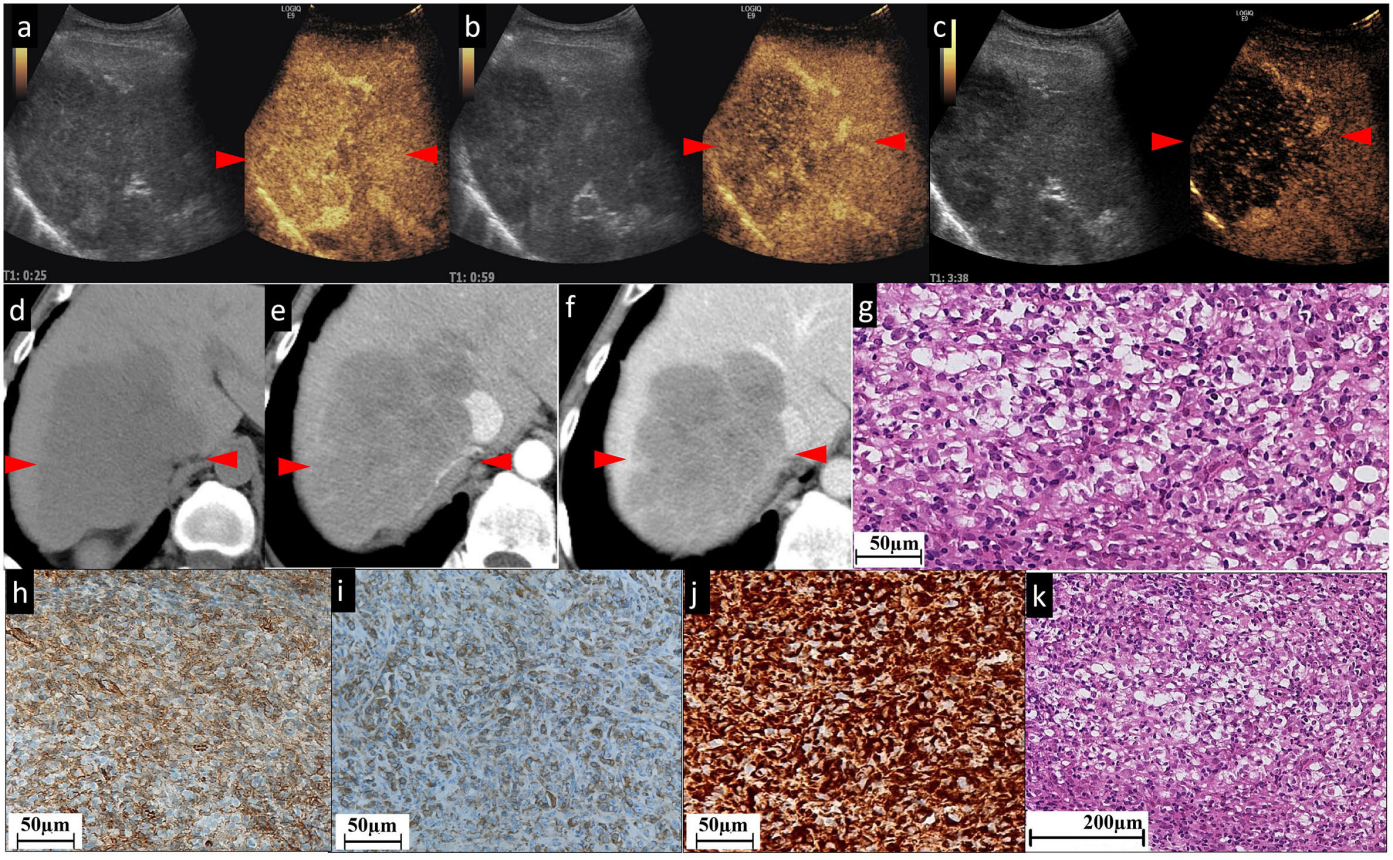
The CEUS and CECT images and histopathological picture of patient No. 3. **(A–C)** The contrast agent is filled unevenly during the whole period of CEUS. A few patchy strip areas or cord-like hyperenhancement are found in AP **(A)**; these patchy strip areas are of continuous hyperenhancement in PP **(B)** and washout (hypoechogenicity) in delayed phase **(C)**. Unenhanced CT **(D)** exhibited that in the right lobe of the liver, there is a low-density mass approximately 111×75 mm in size with clear boundary, irregular shape, and inhomogeneous low internal density. In the contrast-enhanced scan, the whole lesion demonstrates slight **(E)** and persistent **(F)** enhancement, and the border and septal-like areas are more obviously enhanced. In particular, the line-like enhancement at the edge of the lesion indicated by the green arrowhead in **(E)** is a small branch of the coeliac axis. **(G)** and **(K)** Histopathological examination with HE staining showing focally disrupted hepatic architecture with pleomorphic cell malignancy within disrupted sinusoids. Figures **(H, I)** show nodular areas with plenty of CD31 **(H)**- and CD34 **(I)**-positive neoplastic cells. **(J)** Diffuse vimentin expression shows a large number of mesenchymal cells. The red arrowheads seen in **(A–F)** indicate the border of the lesion.

**Figure 4:**
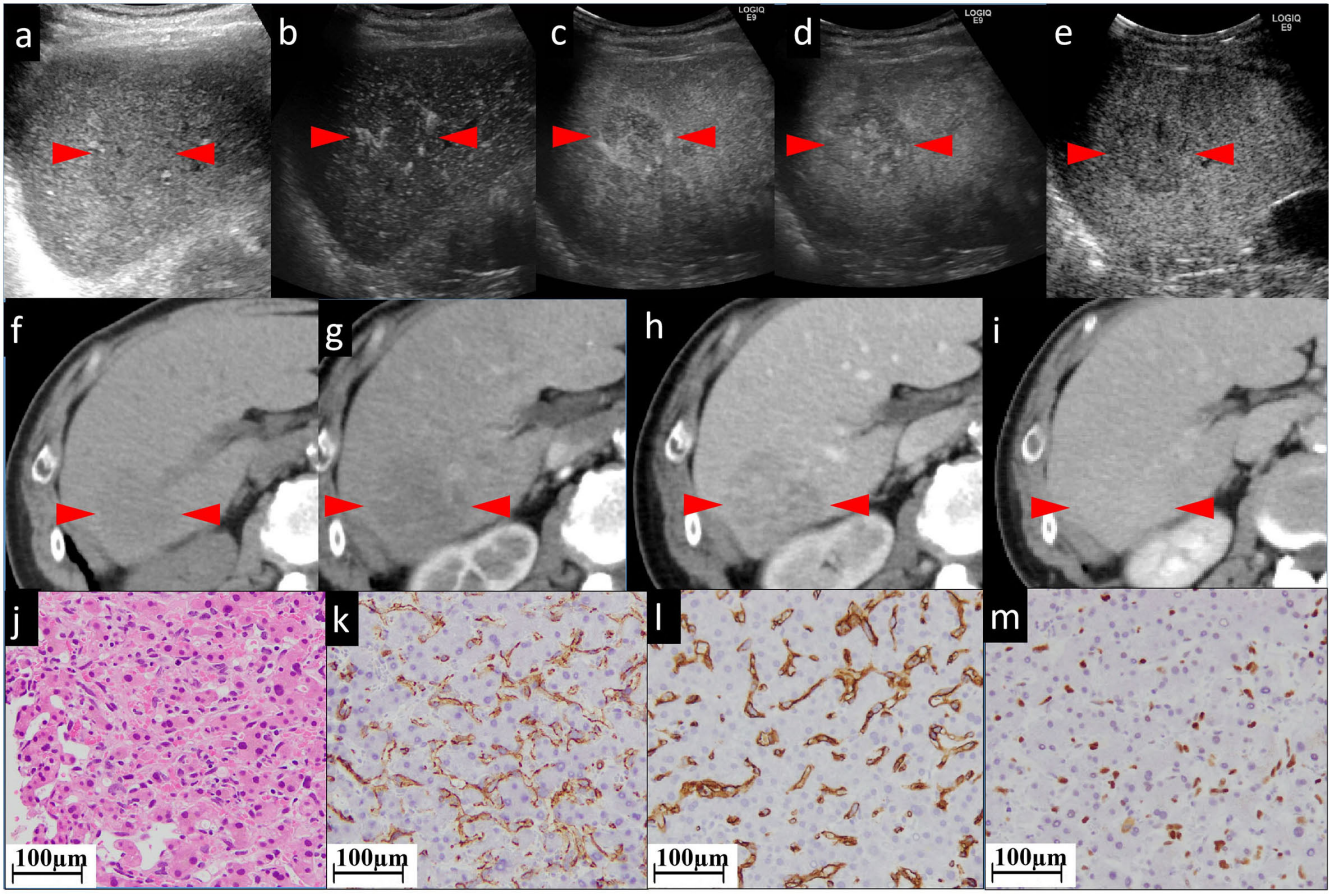
The CEUS and CECT images and histopathological picture of patient No. 4. **(A)** The biggest lesion, located in the right lobe, appears as heterogeneous and hypoechoic, with an ill-defined border in the grayscale US. **(B, C)** In early and late AP of CEUS, the lesion is partially hyperenhanced. From PP **(D)** to postvascular phase **(E)**, the perfusion of the agent gradually decreased. The lesion appears as iso to hypoenhanced. In unenhanced CT **(F)**, the lesion appears as a low-density mass approximately 45×41 mm in size with unclear boundary, irregular shape, and inhomogeneous, slightly high internal density **(G)**. **(F)** shows unenhanced CT. The lesion has an unclear border. In AP **(G)** of enhanced CT, the lesion shows patchy enhancement, while in PP **(H)** it shows persistent fill-in. In the postvascular phase **(I)**, the lesion shows decreased enhancement. Histopathological examination with HE staining **(J)** shows endothelial cells are fusiform, and the nuclei are heavily stained. This is a pathologically early stage of hepatic angiosarcoma. Neither hepatocytes nor sinusoids have been completely destroyed. Tumor cells with nuclei of various sizes (nuclear heteromorphism) grow along the sinusoids. The staining of CD31 **(K)**, CD34 **(L)**, and P53 **(M)** is focally positive. The red arrowheads seen in **(A–I)** indicate the border of our target lesion.

As seen in **Table 1**; **Figures 1**–**4**, and **Supplementary Figures 2**, **3** (as the journal have a maximum limit of no more than four figures for case report, we put the images of patients No. 5 and 6 in supplementary documents), all these lesions appeared as hypoechoic in the US images. The US images revealed the lesions had irregular shapes, unclear borders, and heterogeneous internal echogenicity. No capsule was seen. In the case of multiple nodules, the imaging features observed on the US were identical and, therefore, considered to be of the same nature. In that case, only the largest lesions were observed during the CEUS examination. The mean size of the largest lesion from every patient was 9.2 cm. All the observed lesions were located in the right hepatic lobe. Most HAs (four out of six, 66.7%) in our study appeared as multiple nodules of large size. All the lesions showed an inhomogeneous perfusion of the contrast agents. The lesions consistently exhibited CEUS characteristics such as ill-defined borders and partial hyperenhancement in the AP, while appearing as hypoenhancement in the last phase. The wash-in (in AP) and washout (in the last phase) areas of the contrast agents were exhibited as linear, septal-like, patchy, and scattered shapes.

**Table 1:** US and CEUS features of six enrolled HA lesions ^1^.

Patient Number	US characteristics			CEUS characteristics						
	Echogenicity	Tumor border	Homogeneity	Contrast agent	Perfusion level in AP	Perfusion level in PP	Perfusion level in the last phase	Homogeneity	Tumor border	Dynamic enhancement patterns
No. 1	Hypoechoic with multiple internal patchy, hyperechoic areas	Ill-defined	Heterogeneous	SonoVue	Local hyperenhancement	Hyperenhanced	Hypoenhanced	Heterogeneous	Ill-defined	Multiple patchy internal perfusions and many large areas of perfusion defects
No. 2	same^2^	same^2^	same^2^	same^2^	same^2^	same^2^	same^2^	same^2^	same^2^	Peripheral, slightly linear perfusion and most areas have perfusion defects; no perfusion defect is detected
No. 3	same^2^	same^2^	same^2^	same^2^	same^2^	Hypoenhanced	same^2^	same^2^	same^2^	Multiple patchy internal perfusions and most areas display hypoperfusion
No. 4	Hypoechoic	same^2^	same^2^	Sonazoid	same^2^	Hypo- to isoenhanced	same^2^	same^2^	same^2^	Scattered perfusion and most areas display hypoperfusion
No. 5	Hypoechoic with multiple patchy, hyperechoic areas	same^2^	same^2^	same^2^	same^2^	Hypoenhanced	same^2^	same^2^	same^2^	Irregular linear perfusion and most areas display hypoperfusion
No. 6	same^2^	same^2^	same^2^	same^2^	same^2^	same^2^	same^2^	same^2^	same^2^	same^2^

^1^ HA, hepatic angiosarcoma; US, ultrasound; CEUS, contrast-enhanced ultrasound; AP, arterial phase; PP, portal venous phase.

^2^ “Same” here means “this index is exactly the same as the above row of the same column”.

### 3.3 CECT examination

According to **Table 2**; **Figures 1**–**4**, and **Supplementary Figures 2**, **3**, all the lesions displayed a similar appearance as inhomogeneous low-density masses in unenhanced CT. In the AP, these lesions showed slight enhancement (three out of six, 50%) or absolutely no enhancement (three out of six, 50%) in most areas, while displaying focal areas of marked (four out of six, 66.7%) or mild enhancement (two out of six, 33.3%) in a few areas with a linear, patchy, or septal-like appearance. The focally enhanced areas of all lesions (six out of six, 100%) had persistent enhancement in the PP.

**Table 2:** CT features of six enrolled HA lesions^1^.

Patient Number	Plain CT				CECT				
	Echogenicity	Tumor border	Shape	Homogeneity	Contrast agent	AP	PP	Homogeneity	Tumor border
No. 1	Low-density	Ill-defined	Irregular	Uneven	Iomeprol (Iomeron®)	moderate enhancement overall, except focal patchy marked enhancement	All persistent enhancement	Heterogeneous	Ill-defined
No. 2	same^2^	same^2^	same^2^	same^2^	Omnipaque (Iohexol®)	no enhancement overall, except peripheral, little, thin, linear marked enhancement	Persistent enhancement of linear area	same^2^	same^2^
No. 3	same^2^	Well-defined	same^2^	same^2^	Iopamidol (Iopamiro®)	mild enhancement overall, except septal-like marked enhancement	All persistent enhancement	same^2^	same^2^
No. 4	same^2^	Ill-defined	same^2^	same^2^	same^2^	mild enhancement overall, except patchy marked enhancement	All persistent enhancement	same^2^	same^2^
No. 5	same^2^	same^2^	same^2^	same^2^	same^2^	no enhancement overall while peripheral and internal patchy mild enhancement	Persistent enhancement of AP enhancement area	same^2^	same^2^
No. 6	same^2^	same^2^	same^2^	same^2^	same^2^	no enhancement overall, except septal-like mild enhancement	Persistent enhancement of septal-like area	same^2^	same^2^

^1^ HA, hepatic angiosarcoma; CT, computed tomography; CECT, contrast-enhanced computed tomography; AP, arterial phase; PP, portal venous phase.

^2^ “Same” here means “this index is exactly the same as the above row of the same column”.

### 3.4 Histological diagnosis

The histopathological diagnoses of HA were definitive. All the lesions (six out of six, 100%) showed a positive staining of both CD31 and CD34. All the lesions revealed positive expression of Ki67 to varying degrees.

## 4 Discussion

According to the literature, HA often presents as multiple (57%) and large lesions at first diagnosis (average size of 5.4 cm) (15), which is consistent with our findings (Four of the six patients had multiple lesions, and the mean size of the largest lesion was 9.2 cm). HA progresses rapidly in a short period of time. As seen in **Supplementary Table 1** and **Supplementary Figure 1**, the onset of HA is insidious as the symptoms are mild or uncharacterized. When the patients were first diagnosed with HA, the general condition was good and liver function was normal, similar to BCLC stage B of hepatocellular carcinoma (HCC). However, the condition of patients suffering from HA would deteriorate rapidly. Patients with HA have a much shorter survival [the average survival time documented in the literature review was only 4–6 months (1, 16, 17) while in our study it was 9.3 months) than those with HCC (21.8 months for BCLC stage B (18) and median overall survival of 30 months for all stage of HCCs (19)]. Because the treatment options for HA are not clear and international consensus/guidelines do not exist, the reported treatment options were diverse. Hepatectomy (20), transcatheter arterial chemoembolization (21), local ablation, and liver transplantation (22) were reported to be used. Groeschl, R.T. et al. reported that patients with HA who underwent surgical resection survived longer than those who were untreated (22). For example, Liang J, et al. reported that patients who received aggressive treatment survived 6 to 18 months (20). Lin YC, et al. described cases that survived for 2 years after chemotherapy (21). As for our study, the average survival time of the Chinese patients (who received aggressive treatment) was longer (11.3 months) than that of the Japanese patients (who received only “best supportive care”) (7.3 months). Although large sample data and statistical analyses are still needed to confirm the effectiveness of aggressive treatment, this does indicate the importance of early and accurate diagnosis of HA. Early and accurate diagnosis of HA may give patients the opportunity to receive aggressive treatment, thus benefiting from having a longer survival.

In this study, both CECT and CEUS were used to diagnose HA. Regarding the CEUS examination, all six observed HA lesions in this study appeared as partially hyperenhanced (the areas of hyperenhancement were linear, septal-like, patchy, and scattered) in the AP, and washed out in the last phase. We considered this feature in the CEUS images to be well explained by the histogenesis of HA. The hyperenhancement in the pathologically early stage HA using CEUS might result from the double blood supply of the hepatic artery and portal vein, which was believed to be one cause of HA blood turbulence (23). The relatively slow tumor cell infiltration into the portal vein areas coupled with the rapid sinusoidal tumor cell infiltration led to increased blood supply to the tumor by both the hepatic artery and portal vein (23). CT during arterial photography (CTAP) of HA showed heterogenous contrast enhancement, which suggests blood supply from the portal vein into the masses (24). With regard to the late washout in the last phase, it might be related to the presence of an “arteriovenous short circuit”, portal fistula (25, 26), or even sinusoids dilated in HA. This view has been confirmed by the light microscopic observation of cases both in our study (**Figures 1M**, **2G**) and in the literature (27); there were many disordered, dilated vascular lumina anastomosed to each other in HA lesions, which resulted in the retention and delayed excretion of contrast agents in the vascular systems of the HA lesions. Another characteristic of HA in the CEUS images is that all the lesions were found to be heterogeneously enhanced, with more or less enhancement. Some researchers considered the perfusion defect areas to be an intratumoral hemorrhage, necrosis, and calcifications caused by necrosis (13). When we explore deeper into the disease itself, we prefer to attribute this phenomenon to injury and obstruction of the sinusoids. HA is widely recognized as a tumor with rich blood vessels, especially sinusoids. Normally, slow blood flow passes through sinusoids. In HA, some sinusoids are pathologically dilated with increased pressure and, thereafter, the velocity of blood flow (the aforementioned reason for the hyperenhancement in CEUS). More importantly, many other sinusoids are pathologically obstructed by tumor cells, leading to multiple microthromboses in sinusoid-like structures. It has been reported that the focal hepatic sinusoid injuries caused by chemotherapy have the characteristics of wash-in and washout patterns in CEUS, which are quite similar to those of hepatic metastatic carcinoma (28). Ha F.S., et al. have described a case of HA manifesting as hepatic sinusoidal obstruction syndrome (29). These findings partly explain that the perfusion heterogeneity of contrast agents in CEUS is due to sinusoidal disorders.

The cases in our study showed that CECT and CEUS have many similarities in demonstrating the image features of HA. It is not surprising as the general mechanism of enhanced imaging diagnosis is the contrast agent simulates hemodynamic changes of lesions and makes these changes appear on the image. In detail, in cases No. 1 and No. 4, both CEUS and CECT showed multiple patchy enhancement patterns; in case No. 2, we noted both thin linear enhancement at the edge of the lesion and no enhancement in most areas; and in cases No. 3 and No. 6, both showed septal-like enhancement patterns in the inner and peripheral regions. Although HAs are considered to be variable in images (30), the consistency of two contrast-specific imaging methods, CEUS and CECT, enhances our confidence in using enhanced imaging to diagnose HA.

Based on our imaging findings, we compared the difference between CEUS and CECT, and especially their advantages and disadvantages in the diagnosis of HA.

CEUS is better than CECT in the following respects. First, the gas-filled microbubbles used in CEUS are similar to but slightly different from the other contrast agents used in CT scans. The microbubble contrast agents used in CEUS are bigger than those used in CECT and thus are confined to the intravascular space, making them true blood-pool vascular imaging agents (31). Therefore, the CEUS examination can directly evaluate the microcirculation of lesions. From this perspective, CEUS is presupposed to be more accurate than CECT in diagnosing lesions of vascular origin, such as HA. Second, in our case, CECT and CEUS revealed slight differences in the enhancement of the lesion. Taking the No. 1 case in our study as an example, CECT tended to demonstrate fewer enhancement areas than CEUS in the AP (**Figure 1**). For the No. 2 case, as most areas have no enhancement at all, CEUS appears to present finer linear hyperenhancement at the tumoral border than CECT (**Figure 2**). We think this may be because CECT has fixed predefined but not very accurate time points (32). The transient enhancement, such as that relating to arteriovenous fistula, in the very early AP may have been missed. Late AP in CECT might be mistimed as PP (33). Because of this defect, the very early or late enhancement pattern of hepatic lesions in AP might be undetected or misjudged (6). CEUS does not have this problem because it operates in real-time, that is, continuous imaging with high temporal resolution over the whole enhancement period (32).

However, the relationship between the lesion and the feeding/drainage vessel can be more accurately observed using CECT than CEUS. For example, in case No. 1, the hepatic vein could be seen entering the lesion and thinning locally (**Figures 1G, H**), suggesting an invasion of the hepatic vein by the lesion. In case No. 3, the celiac trunk was found to be circumferential close around the lesion, but continuous and intact (**Figure 3E**). In comparison, these phenomena were not obtained using CEUS in either case, because in the AP of CEUS, the operator should hold the transducer stationary over the area of interest (34), where the lesion can be presented in the largest possible size and with optimal visualization on the scan plane. In this setting, it is possible to not observe the plane where the vein runs. Another problem that should be addressed is that HA has been reported to frequently appear as multiple or dominant lesions (35). Nevertheless, only one lesion can be observed in the AP of CEUS.

The limitations of our study should be acknowledged. First, although this study collected the largest number of cases of HA reported to date based on CEUS examination, the sample size was still small. Therefore, statistical analysis of imaging characteristics was unavailable. Second, the characteristics of CECT and CEUS extracted in this study were visually evaluated by our researchers. No quantitative analyses such as peak contrast intensity or signal intensity were performed, so a certain subjectivity may exist. Third, because our CEUS and needle biopsies were performed only on the largest of multiple lesions, this study did not involve an investigative analysis of the CEUS characteristics of small lesions. Finally, it is difficult to make a more in-depth pathological classification according to the growth pattern of HA because of the small amount of tissue obtained during a biopsy.

## 5 Conclusions

We found that the six cases in this study had some common characteristics in the diagnosis of HA. In CECT, the lesions in the AP showed slight enhancement or no enhancement in most areas, with obvious enhancement of some cord-like, septal-like, or linear structures in the lesions. The enhancement was persistent in the PP. CEUS demonstrated that most areas of the lesions had perfusion defects or sustained low perfusion of contrast agents. There were linear, septal-like, patchy, and scattered structures in the lesions, showing wash-in in the AP and washout in the last phase. In conclusion, we believe that CEUS and CECT are feasible for the preoperative diagnosis of HA.

## Data availability statement

The raw data supporting the conclusions of this article will be made available by the authors, without undue reservation.

## Ethics statement

The studies involving humans were approved by the Research Ethics Committee of The First Affiliated Hospital of Xi’an Jiaotong University (XJTU1AF2023LSK-363) on 6 June 2023 and Yokohama City University Medical Center Institutional Review Board (F220700009) on 27 June 2022. The studies were conducted in accordance with the local legislation and institutional requirements. The participants provided their written informed consent to participate in this study. Written informed consent was obtained from the individual(s) for the publication of any potentially identifiable images or data included in this article.

## Author contributions

FW: Funding acquisition, Writing – original draft. KN: Conceptualization, Writing – review & editing. HL: Formal Analysis, Writing – review & editing. HT: Data curation, Writing – original draft. LR: Supervision, Writing – review & editing. MT: Methodology, Writing – original draft. XB: Investigation, Writing – original draft.

## Supplementary material

Supplementary Figure 1 The timeline of occurrence, diagnosis of hepatic angiosarcoma, treatment, and follow-up in six patients.

Supplementary Figure 2 The CEUS and CECT images and histopathological picture of patient No. 5. In grayscale US image **(A)**, the lesion appears as hypoechoic with multiple patchy, hyperechoic internal areas. It is a pity that the AP and postvascular phase CEUS images have gone missing because of the time elapsed and/or inappropriate saving in the old US system. We can only obtain the CEUS characteristics from the descriptions in CEUS reports. Using a grayscale US image as a reference **(B)**, the lesion shows linear hyperenhancement with multiple patchy internal areas in AP, hypoenhancement as a whole in PP **(C)**, and persistent hypoehancement in the postvascular phase. Most areas of the lesion exhibit perfusion defects throughout the CEUS process. In unenhanced CT **(D)**, the lesion appears as a low-density mass with unclear boundary, irregular shape, and inhomogeneous high internal density. In AP **(E)**, the lesion appears as many small, dotted enhancement patterns and no enhancement in most areas. In PP **(F)**, the previously enhanced dotted area shows persistent fill-in. In the postvascular phase **(G)**, the enhancement is decreased. The black arrows in the HE staining slide **(H)** show that the tumor cells differentiate into blood vessels, forming some malformed lumina. The staining of CD31 **(I)**, CD34 **(J)**, and P53 **(K)** is diffusely positive. The red arrowheads seen in **(A-G)** indicate the border of the lesion.

Supplementary Figure 3 The CEUS and CECT images and histopathological picture of patient No. 6. **(A)** In the grayscale US image, the lesion with the largest size is located in the right lobe, with a diameter of approximately 71 mm and unclear boundary, irregular shape, and inhomogeneous texture. **(B)** is AP of CEUS; the lesion is partially hyperenhanced in a peripheral and septal style. From PP **(C)** to postvascular phase **(D)**, the perfusion of the agent gradually decreased. The previously hyperenhanced area in AP changed to hypoenhanced in the postvascular phase. Most areas of the lesion exhibited perfusion defects throughout the CEUS process. Unenhanced CT **(E)** shows multiple lesions spread over the liver. The biggest one is located in the right lobe, with a size of 92×71 mm and an irregular margin. In AP **(F)**, the biggest lesion exhibits subtle enhancement of the edge and enhancement of the thick septa in the center of the lesion. In the large necrotic area of the lesion, there is no enhancement. In PP **(G)**, the previously enhanced peripheral and septal areas display persistent enhancement. In the postvascular phase **(H)**, the enhancement of the previously enhanced area is decreased. Histopathological examination with HE staining **(I)** shows a proliferation of single-layered tumor cells along sinusoid-like vascular channels with variable degrees of vascular dilatations (black arrows) and atrophy of the intervening liver cell plates. The staining of CD31 **(J)**, CD34 **(K)**, and P53 **(L)** is focally positive. In particular, the tumor cells have highly atypical nuclei. The red arrowheads seen in **(A-I)** indicate the border of our target lesion.
