# Supplementary figures and images for: Case Report: The value of contrast-enhanced ultrasound and contrast-enhanced computed tomography in the diagnosis of hepatic angiosarcoma
Source: Front Oncol. 2023 Dec 1;13:1283544. doi: 10.3389/fonc.2023.1283544 (PMC10722152; doi:10.3389/fonc.2023.1283544)

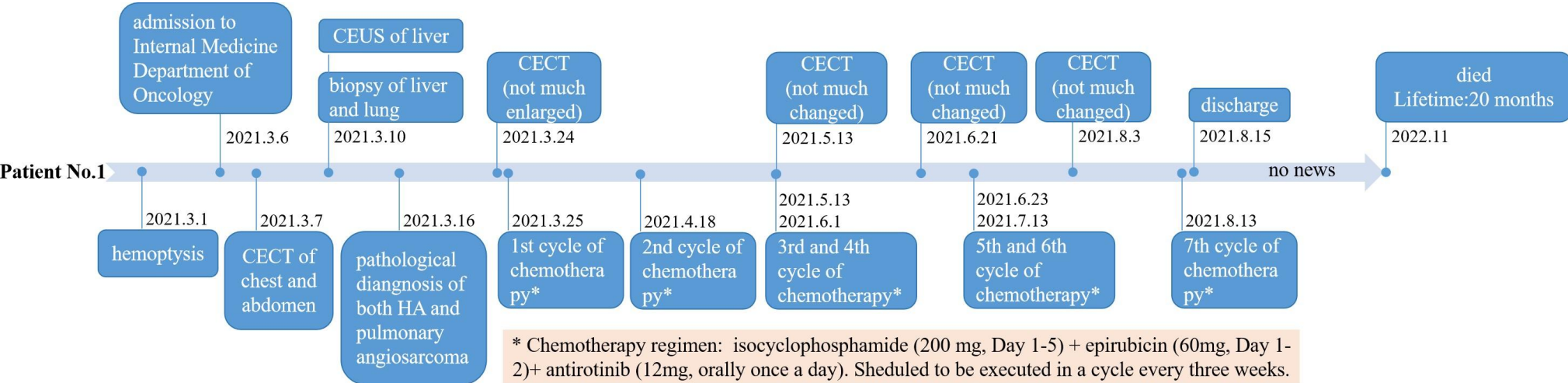

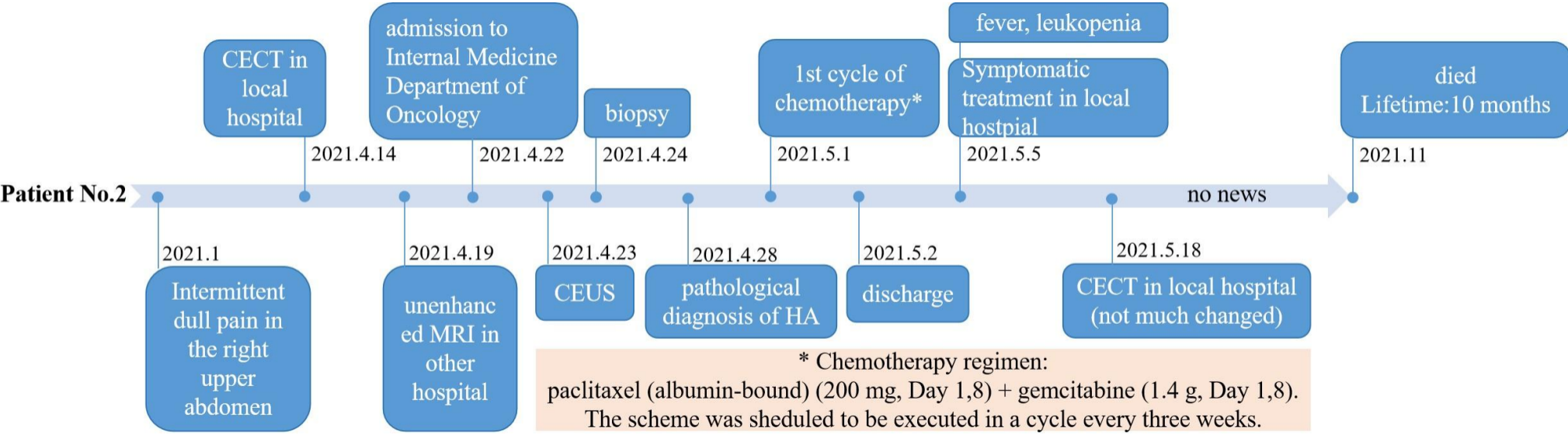

**Patient No.3**

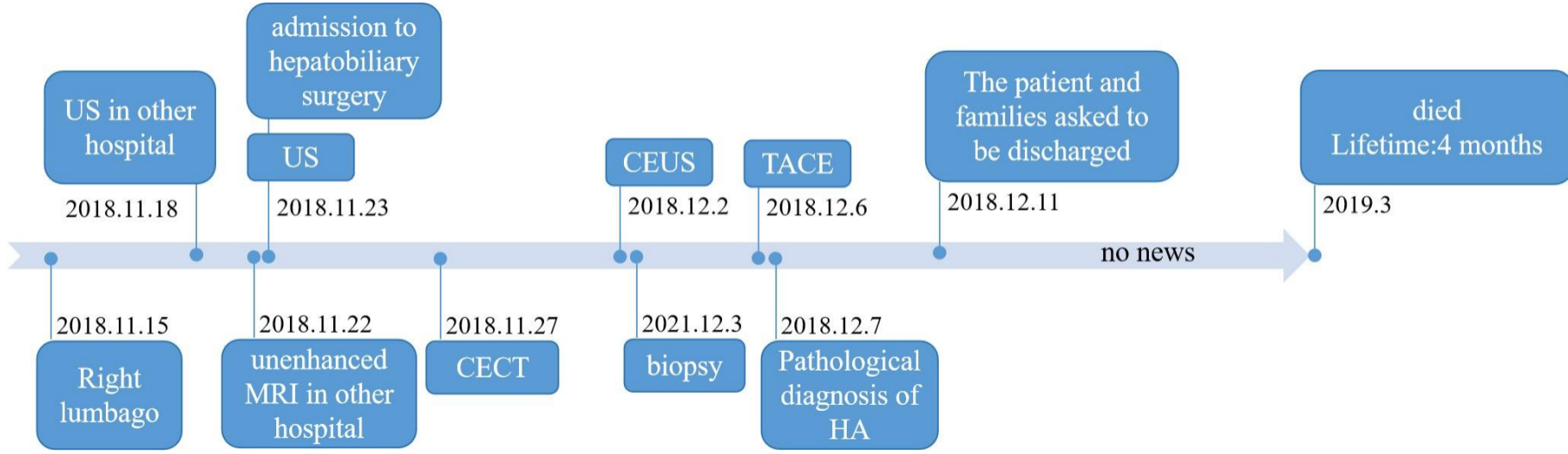

**Patient No.4**

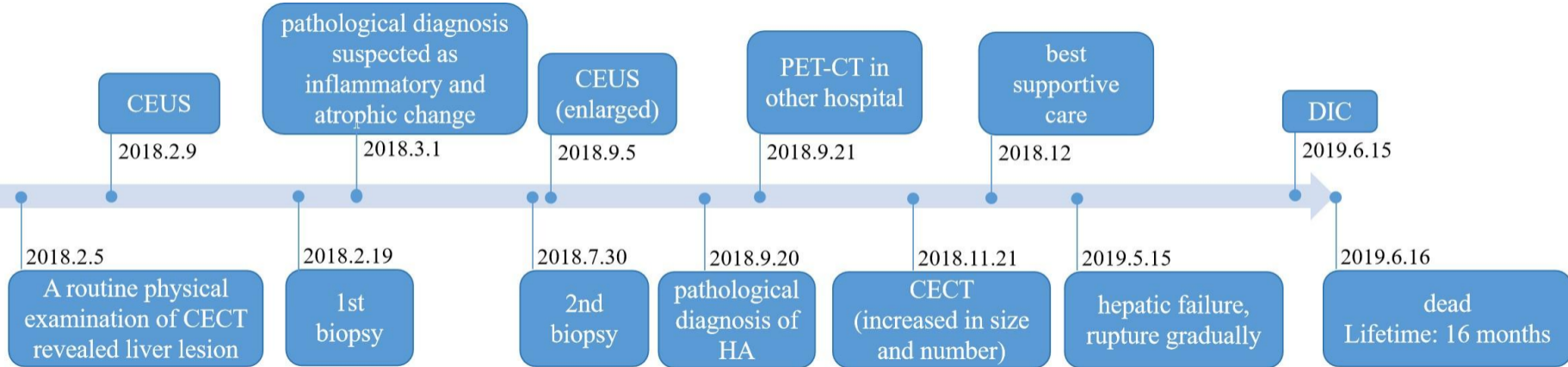

**Patient No.5**

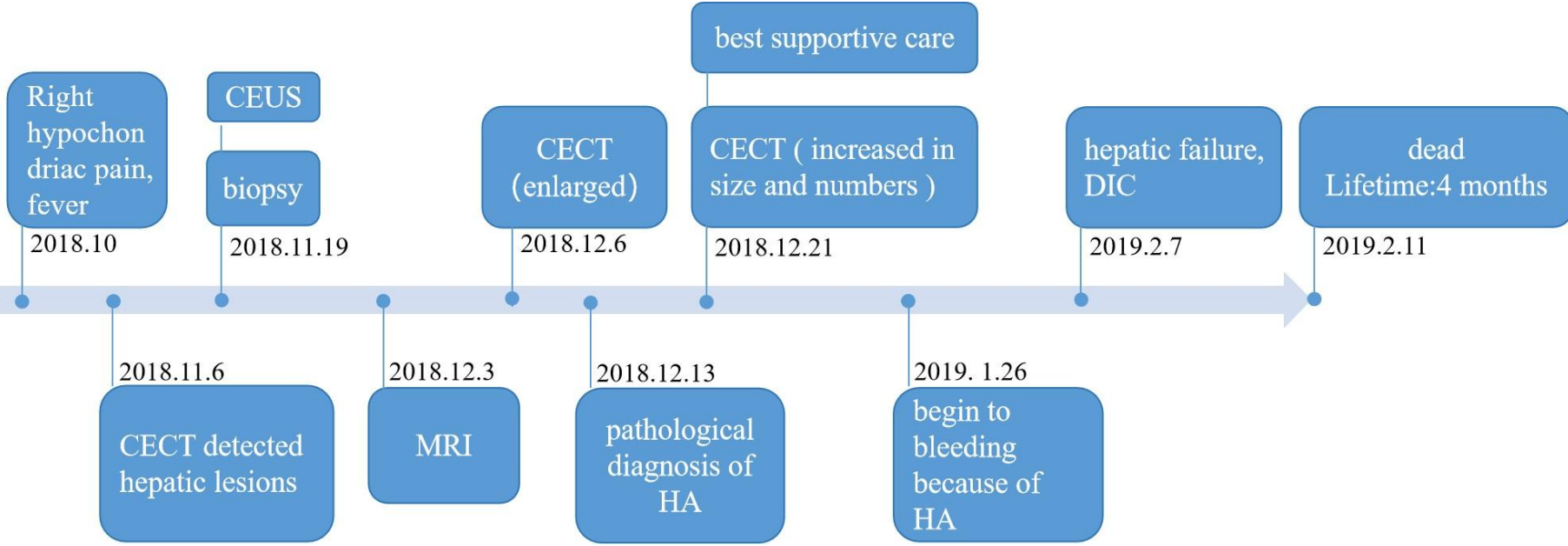

**Patient No.6**

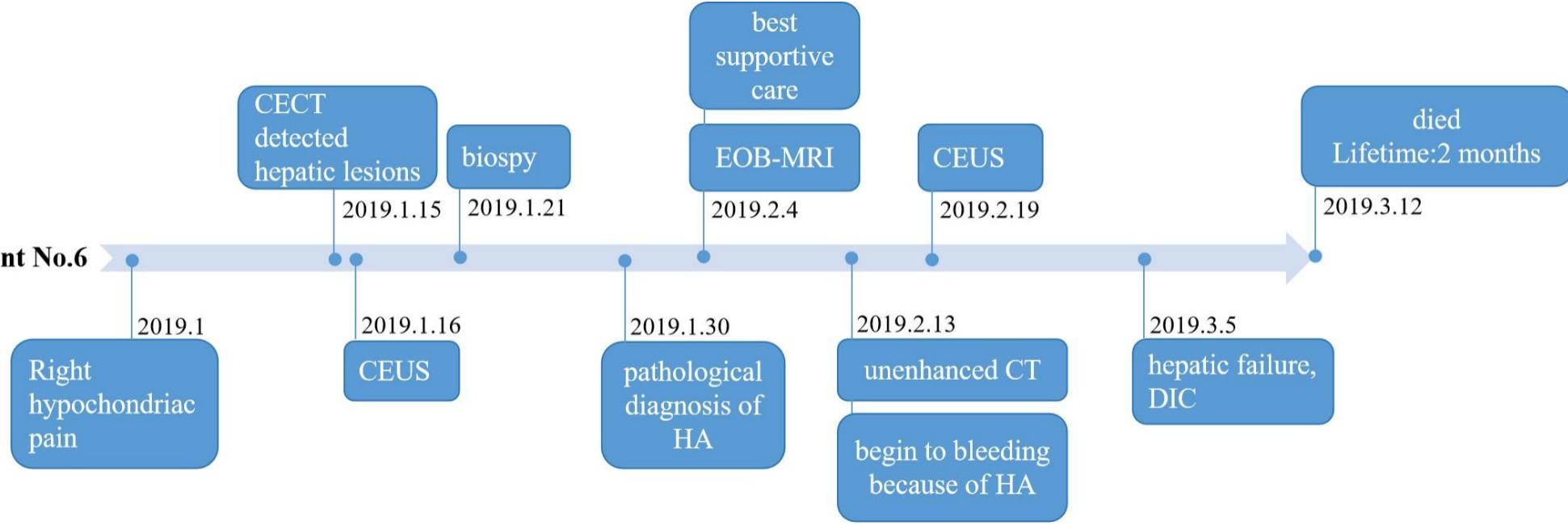

Supplement: Supplementary Figure 1 — The timeline of occurrence, diagnosis of hepatic angiosarcoma, treatment, and follow-up in six patients. [file Image_1.pdf]

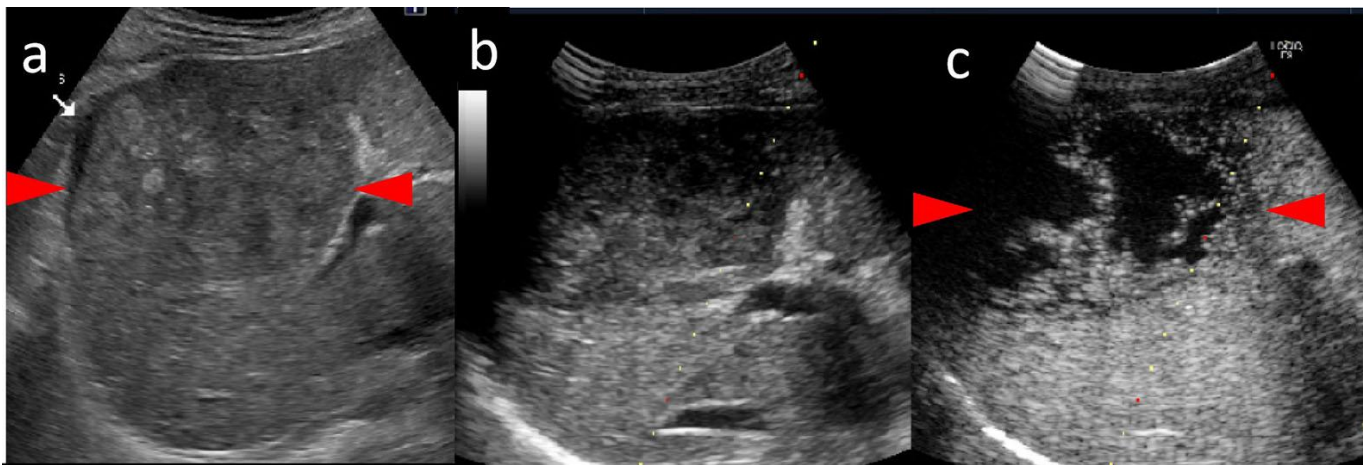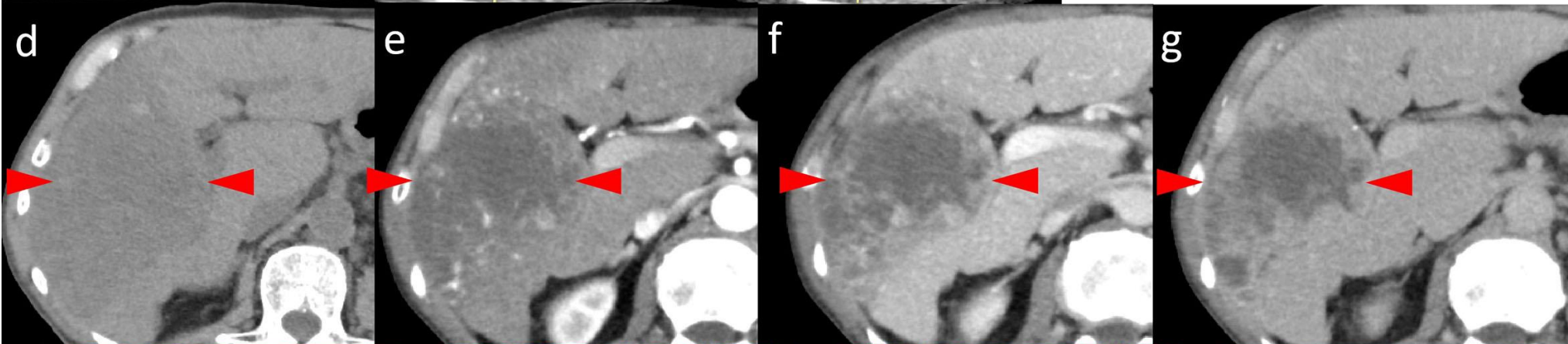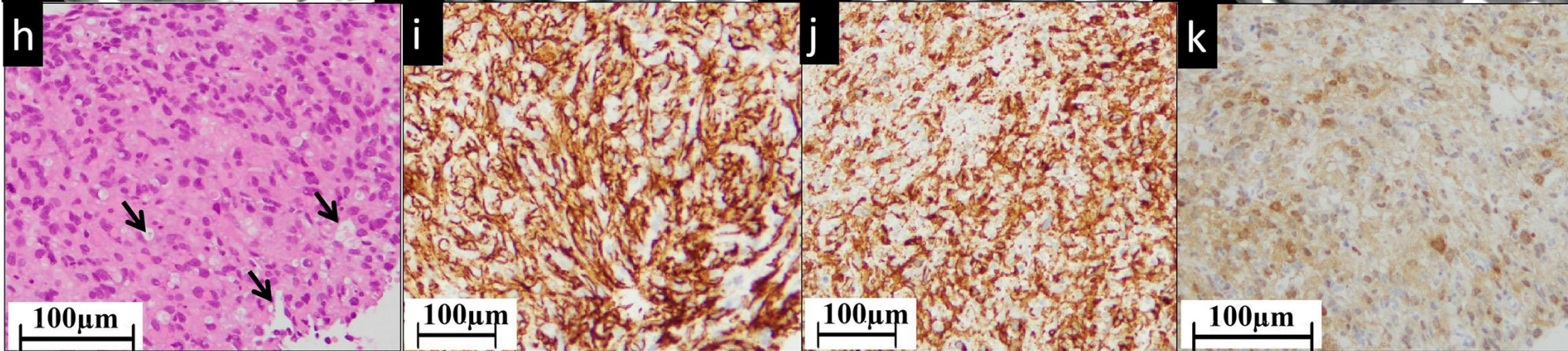

Supplement: Supplementary Figure 2 — The CEUS and CECT images and histopathological picture of patient No. 5. In grayscale US image (A), the lesion appears as hypoechoic with multiple patchy, hyperechoic internal areas. It is a pity that the AP and postvascular phase CEUS images have gone missing because of the time elapsed and/or inappropriate saving in the old US system. We can only obtain the CEUS characteristics from the descriptions in CEUS reports. Using a grayscale US image as a reference (B), the lesion shows linear hyperenhancement with multiple patchy internal areas in AP, hypoenhancement as a whole in PP (C), and persistent hypoehancement in the postvascular phase. Most areas of the lesion exhibit perfusion defects throughout the CEUS process. In unenhanced CT (D), the lesion appears as a low-density mass with unclear boundary, irregular shape, and inhomogeneous high internal density. In AP (E), the lesion appears as many small, dotted enhancement patterns and no enhancement in most areas. In PP (F), the previously enhanced dotted area shows persistent fill-in. In the postvascular phase (G), the enhancement is decreased. The black arrows in the HE staining slide (H) show that the tumor cells differentiate into blood vessels, forming some malformed lumina. The staining of CD31 (I), CD34 (J), and P53 (K) is diffusely positive. The red arrowheads seen in (A-G) indicate the border of the lesion. [file Image_2.pdf]

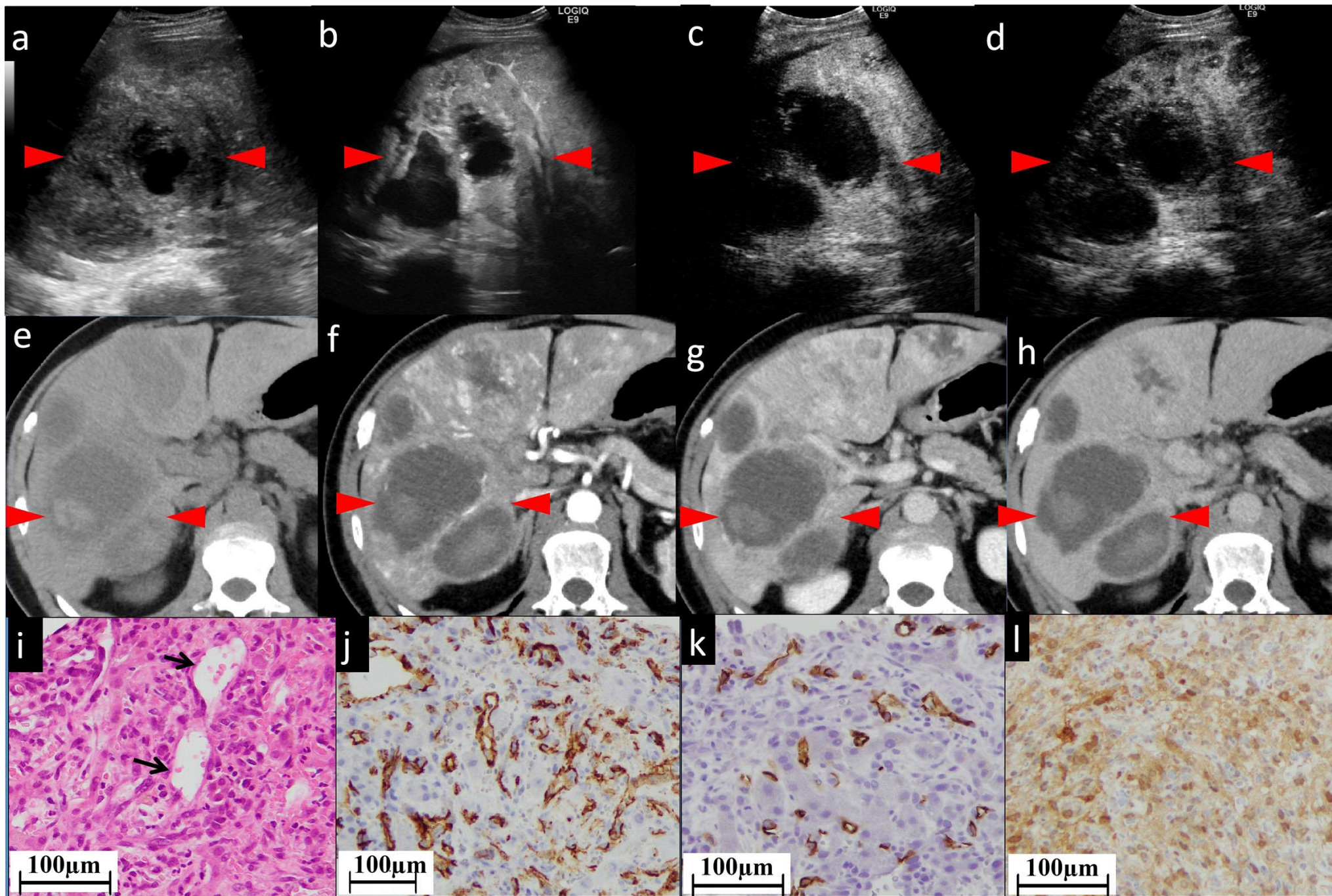

Supplement: Supplementary Figure 3 — The CEUS and CECT images and histopathological picture of patient No. 6. (A) In the grayscale US image, the lesion with the largest size is located in the right lobe, with a diameter of approximately 71 mm and unclear boundary, irregular shape, and inhomogeneous texture. (B) is AP of CEUS; the lesion is partially hyperenhanced in a peripheral and septal style. From PP (C) to postvascular phase (D), the perfusion of the agent gradually decreased. The previously hyperenhanced area in AP changed to hypoenhanced in the postvascular phase. Most areas of the lesion exhibited perfusion defects throughout the CEUS process. Unenhanced CT (E) shows multiple lesions spread over the liver. The biggest one is located in the right lobe, with a size of 92×71 mm and an irregular margin. In AP (F), the biggest lesion exhibits subtle enhancement of the edge and enhancement of the thick septa in the center of the lesion. In the large necrotic area of the lesion, there is no enhancement. In PP (G), the previously enhanced peripheral and septal areas display persistent enhancement. In the postvascular phase (H), the enhancement of the previously enhanced area is decreased. Histopathological examination with HE staining (I) shows a proliferation of single-layered tumor cells along sinusoid-like vascular channels with variable degrees of vascular dilatations (black arrows) and atrophy of the intervening liver cell plates. The staining of CD31 (J), CD34 (K), and P53 (L) is focally positive. In particular, the tumor cells have highly atypical nuclei. The red arrowheads seen in (A-I) indicate the border of our target lesion. [file Image_3.pdf]
