# Supplementary material for: Case Report: The value of contrast-enhanced ultrasound and contrast-enhanced computed tomography in the diagnosis of hepatic angiosarcoma
Source: Front Oncol. 2023 Dec 1;13:1283544. doi: 10.3389/fonc.2023.1283544 (PMC10722152; doi:10.3389/fonc.2023.1283544)
Supplement: Supplementary file 4 [file Table_1.docx]

**Supplementary Table 1. The contrast agents, operating methods, and setting conditions of CEUS^1^**

| **Hospital** | **Type of contrast agent** | **Dose of contrast agent** | **Operation method** | **Mechanical index in CEUS** |
| --- | --- | --- | --- | --- |
| Chinese hospital (for patients No.1,2, and 3) | SonoVue  (Bracco, Milan, Italy) | 2.4 mL | bolus injection into an antecubital vein via a 20-gauge cannula, followed by 5 mL saline flush | <0.2 |
| Japanese hospital (for patients No. 4,5, and 6) | Sonazoid  (GE Healthcare, Milwaukee, WI, USA) | 0.2 mL | bolus injection into an antecubital vein via a 24-gauge cannula, followed by 2 mL of 5% glucose | 0.2–0.3 |

^1^ CEUS—contrast-enhanced ultrasound;

**Supplementary Table 2. Clinical characteristics and pathological findings of six enrolled patients and their HA lesions ^1^**

| **Patient number** | **Age (year) ^2^** | **Gender** | **Symptom** | **Single or multiple** | **Location of hepatic lobe** | **Tumor size (cm) ^3^** | **Etiology** | **Positive tumor markers ^4^** | **Indicators of bleeding, coagulation and anemia** | **BIL** | **CD31** | **CD34** | **Ki67** | **Other positive IHC** |
| --- | --- | --- | --- | --- | --- | --- | --- | --- | --- | --- | --- | --- | --- | --- |
| No. 1 | 60s | Female | Hemoptysis (a lung tumor diagnosed) | Multiple | Right | 12 | No hepatitis | CEA↑ (5.7) | PT:slightly↑ | Direct.BIL↑ | + | + | +10% | Vimentin(+), SMA(±) |
| No. 2 | 60s | Male | Epigastric pain | Multiple | Bilateral | 10 | same^5^ | CEA↑ (6.1) CA19-9↑ (46) | PLT:slightly↓  HGB:slightly↓ | Direct.BIL↑ Total.BIL↑ | same^5^ | same^5^ | +80% | Vimentin(+), ERG(+) |
| No. 3 | 60s | same^5^ | Right lumbago | Single | Right | 11 | same^5^ | CEA↑ (3.5) CA125↑(80) CA19-9↑(48) | HGB:slightly↓ | All (–) | same^5^ | same^5^ | +80% | Vimentin(+), |
| No. 4 | 70s | same^5^ | Asymptomatic liver tumor | Multiple | same^5^ | 4.2 | PBC | All(–) except CA125 undone | Hb:markedly↓ | same^5^ | same^5^ | same^5^ | +＞  20% | P53(+), |
| No. 5 | 60s | same^5^ | Right hypochondriac pain, fever | Single | same^5^ | 8.5 | No hepatitis | All(–) except CA125 undone | Hb:slightly↓  PLT:markedly↓ | same^5^ | same^5^ | same^5^ | +＞  40% | P53(+) |
| No. 6 | 50s | same^5^ | Right hypochondriac pain | Multiple | Bilateral | 9.2 | same^5^ | CA19-9↑(62) | All (–) | same^5^ | same^5^ | same^5^ | +＞30% | SMA(+), P53(+) |

^1^ HA—hepatic angiosarcoma; CA125—carbohydrate antigen 125; CA19-9—carbohydrate antigen 199; CD31—cluster of differentiation antigen 31; CD34—cluster of differentiation antigen 34; SMA—smooth muscle actin; PLT—platelet count; PT—prothrombin time; HGB—hemoglobin; PBC—primary biliary cholangitis; BIL—bilirubin; ERG—erythroblast transformation-specific (ETS)-related gene.

2 We did not describe the exact age of the patients to ensure that the patients are not identified because of the exposure of personal details.

3 For multiple lesions, the size written in the above table indicates the largest lesion.

4 The tumor markers include AFP (alpha-fetoprotein), CA125, CA19-9, and CEA (carcinoembryonic antigen).

5 “Same” here means “this index is exactly the same as the above row of the same column”.
